# Multisensory processing impacts memory for objects and their sources

**DOI:** 10.3758/s13421-024-01592-x

**Published:** 2024-06-03

**Authors:** Shea E. Duarte, Andrew P. Yonelinas, Simona Ghetti, Joy J. Geng

**Affiliations:** 1 Department of Psychology, University of California, Davis, CA 95616 USA; 2 Center for Mind and Brain, University of California, Davis, CA 95618 USA; 3 Center for Neuroscience, University of California, Davis, CA 95618 USA

**Keywords:** Multisensory memory, Recognition, Source memory, Audiovisual processing

## Abstract

Multisensory object processing improves recognition memory for individual objects, but its impact on memory for neighboring visual objects and scene context remains largely unknown. It is therefore unclear how multisensory processing impacts episodic memory for information outside of the object itself. We conducted three experiments to test the prediction that the presence of audiovisual objects at encoding would improve memory for nearby visual objects, and improve memory for the environmental context in which they occurred. In Experiments 1a and 1b, participants viewed audiovisual–visual object pairs or visual–visual object pairs with a control sound during encoding and were subsequently tested on their memory for each object individually. In Experiment 2, objects were paired with semantically congruent or meaningless control sounds and appeared within four different scene environments. Memory for the environment was tested. Results from Experiments 1a and 1b showed that encoding a congruent audiovisual object did not significantly benefit memory for neighboring visual objects, but Experiment 2 showed that encoding a congruent audiovisual object did improve memory for the environments in which those objects were encoded. These findings suggest that multisensory processing can influence memory beyond the objects themselves and that it has a unique role in episodic memory formation. This is particularly important for understanding how memories and associations are formed in real-world situations, in which objects and their surroundings are often multimodal.

**Supplementary information:**

The online version contains supplementary material available at 10.3758/s13421-024-01592-x.

The ability to remember events is fundamental to the human experience. In order to successfully store information into episodic memory, the brain must form novel associations between individual objects (e.g., a sea lion and seagulls) and their context (e.g., San Francisco’s Pier 39; Dickerson & Eichenbaum, 2010; Mitchell & Johnson, 2009; Yonelinas et al., 2019). While memories can be formed for individual objects, episodic memories are unique in that they consist of relational representations including spatial, temporal, and other details of the event in which the objects were encountered to provide a cohesive story about an experience. Laboratory studies of episodic memories have mostly focused on visual stimuli (e.g., seeing a sea lion at Pier 39), but real-world experiences involve information presented through multiple senses at the same time: the sea lion *barks* and the seagulls *squawk*. In contrast, there have been many studies of multisensory object processing, but most of these have focused on the processing of individual objects that appear alone. These studies show that multisensory objects are identified faster, are more likely to be attended and remembered, and facilitate learning (Macaluso et al., 2016; Matusz et al., 2017; Shams & Seitz, 2008; Stein et al., 2020). However, little is known about how multisensory objects impact the formation of episodic memories. The current experiments bring together two literatures to test the hypotheses that the presence of a multisensory object at encoding will improve memory for nearby visual objects and the environmental context.

A number of studies have demonstrated that general recognition memory is better for visual objects encoded with an object-congruent sound (e.g., dog + *bark*) compared with an incongruent sound (e.g., dog + *ding*), a meaningless tone, or no sound at all (Heikkilä et al., 2015, 2017; Lehmann & Murray, 2005; Moran et al., 2013; Thelen & Murray, 2013; see Matusz et al., 2017, for a review). These studies used old/new memory assessments and found better recognition memory performance for objects that were experienced audio-visually compared with only visually. However, successful recognition of “old” objects can be accomplished via two mechanisms that are neurally and behaviorally dissociable: familiarity and recollection (Diana et al., 2007; Eichenbaum et al., 2007; Yonelinas, 2002). Familiarity is a general, strength-based type of recognition memory for individual objects, whereas recollection-based recognition includes details of the unique context in which an item was experienced.

In a recent study, we attempted to determine whether multisensory experiences improved memory through familiarity or recollection. We found that the memory benefits of seeing a visual object with a congruent sound were specific to recollection-based recognition and did not extend to familiarity (Duarte et al., 2022). Further, we found better memory for the sounds played during encoding when those sounds were congruent (compared with incongruent or meaningless sounds). This suggests that an object’s corresponding sound provided an additional route for accessing the object in memory. However, the results were surprising because recollection typically supports memory for details outside of the studied item itself, including the environment in which an object was encoded or other nearby objects (Mitchell & Johnson, 2009). Thus, one open question is whether the recollection-specific memory benefit would extend to other objects or contexts.

The goal of the current research is to build on our previous findings and investigate the effects of multisensory object encoding on memory for neighboring objects and background context. One possibility is that multisensory encoding could improve memory by increasing within-object feature processing of that object itself at a cost to memory for other objects and the context. Studies have shown this trade-off between memory for object and context information when a certain object or feature is selectively attended (e.g., Uncapher & Rugg, 2009). Alternatively, audiovisual objects may promote memory by forming a more elaborate memory representation that includes other objects and contextual details present at encoding. Evidence for this comes from Murray and colleagues (2022), who found that names of faces were better recollected when participants were provided with audiovisual name cues (name tags + spoken names) relative to visual-only cues (written names alone) at encoding. In this case, audiovisual name encoding made it easier to later name the associated face. The primary goal of the present work was therefore to investigate whether, and how, audiovisual object processing affects features of episodic memories beyond the object itself.

Here, we report results from three experiments testing the effect of audiovisual object processing on neighboring visual objects (Experiments 1a & 1b) and source memory for contextual details in which the object was encoded (Experiment 2). In Experiments 1a and 1b, participants viewed audiovisual–visual object pairs or visual–visual object pairs with a control sound, and their memory for each object was tested separately. In Experiment 2, audiovisual objects with semantically congruent or meaningless control sounds were embedded within scenes from one of four different environments, and source memory for the environments was tested. To anticipate our results, we found that encoding a congruent audiovisual object did not significantly improve or impair memory for neighboring visual objects. However, congruent audiovisual objects yielded better source memory for the scene context in which they occurred. Our results suggest that multisensory object processing enhances episodic memory, but selectively for source information.

## Experiments 1a and 1b

**Fig. 1:**
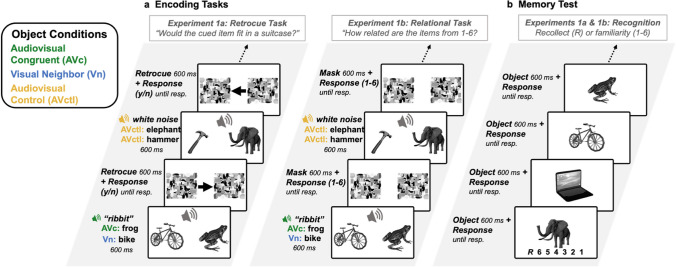
Task design for Experiments 1a & 1b. **a.** The Retrocue Encoding Task used in Experiment 1a (left) in which participants make a size judgement about the object in the retroactively cued location, and the Relational Encoding Task used in Experiment 1b (right), in which participants rate (1-6) how related the two items are. **b.** Surprise visual recognition memory test used in both Experiments 1a & 1b, in which participants indicate that they recollect (R) the object or indicate recognition confidence by selecting “definitely old,” “probably old,” “maybe old,” “maybe new,” “probably new,” or “definitely new.” Confidence levels are denoted in the figure as 6 (definitely old) to 1 (definitely new). (Color figure online)

The main objectives of Experiments 1a and 1b were the same: to test the hypothesis that multisensory object processing supports memory for other visual objects present at encoding. In both experiments (Fig. 1), participants completed a within-subjects audiovisual encoding task in which two objects were shown on each trial with a sound that was presented centrally (i.e., presented from speakers evenly rather than localized to the right or left). Participants were asked to make a judgment based on the two objects shown. At the end of the experiment, participants were given a surprise recognition memory test of the objects seen during the encoding task. The sound presented on each trial was congruent to one of the two objects (e.g., bicycle + frog + *ribbit*) or neither of them (e.g., bicycle + frog + *white noise*). Therefore, the individual objects were encoded as either an Audiovisual Congruent object (the frog + *ribbit*), a Visual Neighbor (the bicycle neighboring the frog + *ribbit*) or an Audiovisual Control (the frog + *white noise*). The Audiovisual Control condition was chosen instead of a condition with two unisensory visual-only stimuli to control for the possibility that the presence of any sound may bolster memory by enhancing the distinctiveness between objects or by generally increasing attentional alerting. Our Audiovisual Control condition was therefore a stricter test of how sounds impact memory for visual objects.

Experiments 1a and 1b were identical other than the type of judgment made during the encoding task. In Experiment 1a, participants made a size judgment about one of the two objects, indicated by a retroactive cue. In Experiment 1b, participants rated the semantic relatedness of the two objects from 1 to 6. These encoding tasks differ in that Experiment 1a asks participants to process the two objects individually, but Experiment 1b asks participants to process the two objects in relation to each another. In both Experiments, we used Receiver Operating Characteristics (ROCs) to examine recollection-based and familiarity-based recognition memory for each object individually after the encoding task. In this recognition test, participants determined whether a single object had been previously presented by indicating whether each item was old or new, along with their confidence (Yonelinas et al., 2010). We predicted better recollection for the Visual Neighbor objects than the Audiovisual Control objects in both experiments. However, if the multisensory memory benefit requires relational processing to extend to nearby objects, then we would expect the Visual Neighbors to show improved memory only in Experiment 1b and not Experiment 1a. Together, these experiments provide two methods of investigating the question of whether audiovisual information about a single object impacts recognition memory for a co-occurring neighboring object during encoding: individual processing (Experiment 1a) and relational processing (Experiment 1b).

### Method

#### Participants

Our sample size for Experiments 1a and 1b was determined by an a priori power analysis using the Python package Pingouin (Vallat, 2018) with power (1 − b) set at 0.95 and *a* = 0.05. Prior data from our lab (Duarte et al., 2022) showed an effect of initial sound congruency on recognition memory for visual items with an effect size of η^2^ = 0.1, which requires at least 25 participants to detect. To account for potentially poor testing conditions associated with online data collection, we doubled this number to 50, and data were collected in each experiment until we reached this number of participants postexclusion. The debriefing questionnaire was used to exclude participants by identifying those who participated in a noisy testing environment, exerted little or no effort in completing the study, lacked access to consistent audio (due to glitches, volume changes, or a lack of working speakers), or misunderstood either task (see Supplemental Materials for full list of questions). The sample size and exclusion criteria were preregistered for Experiment 1a and replicated for Experiment 1b, and can be found on the Open Science Framework (https://osf.io/b3gwt).

##### **Experiment 1a**

One hundred and nine students from the University of California, Davis, participated in exchange for partial course credit, and 50 were included in analyses (34 identified as female, 13 identified as male, and three identified as nonbinary, *M*_age_ = 19.02 years; see Supplemental Materials for full sample demographics). Based on our preregistered exclusion criteria, two participants were excluded from Experiment 1a due to low accuracy on the encoding task (below 50%), and 57 were excluded because they were unable to provide examples demonstrating that they understood when to respond with “recollect.”

##### **Experiment 1b**

Eighty-three students from the University of California, Davis, participated in exchange for partial course credit, and 50 were included in analyses (40 identified as female, eight identified as male, and one identified as nonbinary, *M*_age_ = 19.66 years; see Supplemental Materials for full sample demographics). Seven participants were excluded due to low accuracy on the recognition task (below 50%), and 26 were excluded because they were unable to provide examples demonstrating that they understood when to respond with “recollect.” Participants were ineligible to participate in Experiment 1b if they had participated in Experiment 1a.

#### Materials

Experiments 1a and 1b: A total of 180 images of three-dimensional (3D) models of common objects (e.g., animals, instruments, common household objects; see Supplemental Materials for a full list of items) were gathered from the Unity Asset Store (https://assetstore.unity.com/3d). 3D models were edited in Unity to easy-to-recognize orientations and to reflect the position the objects typically assume when they produce a sound (e.g., the dog model was edited to have an opened mouth, as if it were barking). We used the Python package scikit-image (Van der Walt et al., 2014) to remove the image backgrounds, convert them to black and white, and size them to the same dimensions (to fit within 500 × 500 pixels). The real-world sizes of half of the objects in the images were “small” (small enough to fit in a standard suitcase) and the other half were “large.” Ninety of the objects were used in the encoding task, and all 180 were used in the recognition memory test. New items in the recognition test were selected from the same categories as the old objects (e.g., animals, instruments, common household objects). The old and new items were not counterbalanced across the encoding and recognition tasks, but, importantly, the old items, which all had associated sounds, were counterbalanced across the three encoding conditions across participants. Therefore, while overall recognition discrimination between old and new objects may be different, this would not affect the critical comparisons of interest between recognition of items paired with different sounds in the Audiovisual Congruent, Visual Neighbor, or Audiovisual Control encoding conditions. Six different poststimulus visual masks were manually created using a variety of black, white, and gray geometric shapes arranged in a square the same size as the images.

Natural sounds and white noise sounds were obtained from the Multimost Stimulus Set (Schneider et al., 2008) or found online (https://findsounds.com/). Ninety natural sounds corresponded to the items in the encoding task for the audiovisual items and were selected because the contents were recognizable within the 400-ms duration (see Supplemental Materials for a full list of images and sounds used in each experiment and sound descriptions; see Schneider et al. [2008] for further description of Multimost Stimulus Set stimuli). Fifteen variations of white noise were used for the control condition. All sounds were centrally presented, 400 ms in duration and amplitude normalized using Audacity (Audacity, 2021). Sounds were kept at 400 ms to match the duration from the Multimost Stimulus Set, though it should be noted that this was shorter than the visual stimulus presentation, which was extended to 600 ms to ensure enough time to view both items presented at once. The sounds and images onset at the same time, so there was 200 ms of visual object presentation after the conclusion of each sound.

#### Procedure

Experiments 1a and 1b: Participants completed this study online using the Testable platform (https://www.testable.org/). At the start of each experiment, a string of sample beeps was played, and participants were asked to adjust their sound level to a comfortable volume and not to alter it for the remainder of the study. After general instructions, participants completed separate encoding and recognition tasks, followed by a debriefing questionnaire. For Experiment 1a, participants completed the Retrocue Encoding Task, and in Experiment 1b, participants completed the Relational Encoding Task (see below). The Recognition Memory Test and Debriefing Questionnaire were the same in both experiments. Time-to-complete the experiment was not recorded for each participant, though each experiment took approximately 20 minutes to complete during internal pilot testing.

#### Encoding tasks

##### **Experiment 1a: Retrocue encoding task**

This consisted of a size judgement task in which participants viewed two items on each trial and received a retroactive cue indicating which item they should base their response on (Fig. 1a). Forty-five object pairs were presented during this block, for a total of 90 objects (30 Audiovisual Congruent items, 30 Neighboring Visual items, 30 Audiovisual Control items). This resulted in 30 trials with a meaningful congruent sound and 15 trials with a meaningless sound. This was done to ensure there would be an equal number of individual objects in each Object Condition. On each trial, two objects were presented to the left and right of fixation for 600 ms, along with a centrally presented sound for 400 ms. The sound was semantically congruent with one of the objects or was a meaningless control sound. On the trials with a meaningful sound that matched an item, there was one Audiovisual Congruent object (e.g., frog + *ribbit* in Fig. 1a) and one Visual Neighbor object (e.g., the bicycle in Fig. 1a). On trials with a meaningless sound, there were two Audiovisual Control objects (e.g., the hammer and elephant + *white noise* in Fig. 1a).

After the 600-ms (total) stimulus duration, two poststimulus visual masks appeared in the position of the two visual images to limit continued visual processing of the objects (Kinsbourne & Warrington, 1962). Between the two visual masks was a retroactive cue (retrocue) in the form of an arrow pointing to the left or the right. Participants were instructed to click on a “yes” or “no” button to indicate whether the item that had been in the cued position would fit in a standard-sized suitcase. The objects were paired such that 15 trials had two small objects, 15 trials had two large objects, and 15 trials had one small and one large object to ensure that participants had to identify both objects on each trial to perform well. Participants were also instructed to ignore the sounds. The masks were presented until the participant responded, and there was a 600-ms intertrial interval between each trial (not depicted in Fig. 1a). The items were counterbalanced across Object Conditions, such that every item appeared as an Audiovisual Congruent item, a Visual Neighbor item, or an Audiovisual Control item across three versions of the experiment between participants. The probability of the retrocue pointing to the left or to the right was equal across all trial types and Object Conditions. The retrocue/size judgement task was designed to require participants to identify both visual items during the initial presentation, without specifically freeing them to focus on either individual item, and to prevent participants from anticipating a memory test, making the subsequent recognition block a test of incidental object memory.

##### **Experiment 1b: Relational encoding task**

The encoding task for Experiment 1b consisted of an item pair relation judgement task in which participants view two items on each trial and rate how likely the two objects are to be seen together in the real world from 1 (*rarely/never*) to 6 (*often/always*) (Fig. 1a, right). The encoding block was similar to Experiment 1 in that there were 45 visual object pairs presented during the block, for a total of 90 visual items (30 Audiovisual Congruent items, 30 Neighboring Visual items, 30 Audiovisual Control items). The items in this task were paired such that half of the items were likely to be seen together in the world often or always (e.g., goat & pig), and the other half were likely to be seen together rarely or never (e.g., penguin & microwave). As with Experiment 1, on each trial, two visual items were presented to the left and right of fixation for 600 ms, along with a centrally presented sound for 400 ms. The sound was semantically congruent to one of the visual items, or was a control white noise sound. After the 600-ms stimulus, two poststimulus visual masks appeared in the position of the two visual images, which functioned to limit continued visual processing of the object in order to accentuate the timing co-occurrence of the visual and auditory stimuli (Kinsbourne & Warrington, 1962). During this time, participants indicated, from 1 to 6, how likely these items were to be seen together in the real world. As in Experiment 1a, participants were instructed to ignore the sounds. The masks were presented until the participant responded, and there was a 600-ms intertrial interval between each trial (not depicted in Fig. 1a). As in Experiment 1a, the items were counterbalanced across Object Conditions, such that every item appeared as an Audiovisual Congruent item, a Visual Neighbor item, or an Audiovisual Control item across three versions of the experiment between participants. This task was designed to encourage participants to not only identify both items individually, but to consider their relation to one another to facilitate between-object binding, and to prevent participants from anticipating a memory test.

#### Recognition memory test

Experiments 1a and 1b: This memory test was designed to dissociate between recollection-based and familiarity-based recognition in accordance with the dual-process signal detection model using responses to six response criteria to construct receiver operating characteristics (ROCs; Yonelinas, 1994). However, there are multiple methods for dissociating between recollection-based and familiarity-based recognition, which differ in their assumptions of the characteristics of each process (see Yonelinas et al., 2010). We therefore included a subjective, introspective measure of recollection to allow us to conduct a supplemental analysis using the remember/know (recollect/familiar) procedure to assess whether results converge across multiple process-dissociation methods (Tulving, 1985; see data analysis, Supplemental Materials Appendix A).

In the visual-only memory test, the 90 old images were intermixed with 90 new images for a total of 180 trials. On each trial, a single visual stimulus was presented for 600 ms, and participants could respond by clicking on buttons corresponding to an introspective report of “recollect” or to one of six other response criteria: “definitely old,” “probably old,” “maybe old,” “maybe new,” “probably new,” or “definitely new” (Fig. 1b). Participant instructions included a description and example of the difference between a “recollect” response and any “old” response, explaining that “recollect” should only be pressed if the participant was sure that they had seen the item before *and* they could recollect some qualitative information about the encoding event, such as their feelings about the item or what they thought about when they initially saw it. The item presented on each trial corresponded to one of the three Object Conditions (Audiovisual Congruent, Visual Neighbor, Audiovisual Control) from the encoding task. The order of objects was randomized for each participant.

#### Debriefing questionnaire

Experiments 1a and 1b: After the experiment, participants responded to questions on a debriefing questionnaire, which allowed us to assess the quality of the testing environment and stimulus presentation. This survey included questions about the testing environment, the subjective volume and quality of the auditory stimuli, whether the volume was adjusted during the experiment, whether any glitches or lags between audiovisual stimuli were experienced, among others (see Supplemental Materials Table 4 for a full list of questions). As this experiment was completed remotely, responses to this survey were used to exclude participants when the testing environment or stimulus presentations were not of adequate quality. To ensure that participants understood the recognition task, the debriefing survey included a question asking whether participants understood when they were supposed to press the “recollect” button. We also included a free-response question asking for an example of information they used to judge an item as recollected rather than definitely old on one of the trials. While this question only asked for a single example from each participant, we performed exploratory analyses on these responses to assess the types of details participants found salient enough to mention.

#### Data analysis

The design, hypotheses, and statistical analyses for Experiment 1a were preregistered prior to data collection on the Open Science Framework (https://osf.io/b3gwt), and the same statistical analysis approach was used for Experiment 1b.

Experiments 1a and 1b: We compared overall memory performance for items between Object Conditions using the observed area under the curve (AUC). To directly assess effects of Object Conditions on recollection- and familiarity-based recognition, we fit the Dual-Process Signal Detection (DPSD) model to our confidence data (Yonelinas, 1994). Subjective “recollect” responses were used to perform supplementary remember/know analyses to assess the convergence of our results across multiple recollection and familiarity process-dissociation methods (see Supplemental Materials Appendix A). We also performed an exploratory analysis of responses to the open-ended debriefing survey prompts asking participants to report an example of information they used to base their “recollect” responses on. Additionally, we have included mean hit rate (% correct recognition of old items) and false-alarm rate (% incorrect recognition of new items) across response criteria for the recognition tasks in the Table 1. Performance on the encoding tasks for both Experiments (% correct) are reported in Table 7 of the Supplemental Materials, and exploratory analyses of the relation between encoding and memory performance are reported below. Raw data files for both experiments are publicly available on the Open Science Framework (https://osf.io/sep3r/).

**Table 1:** Mean hit rates (% correct recognition of old items) and false-alarm rates (% incorrect recognition of new items) for the recognition tasks, and mean DPSD model parameters for overall recognition memory (AUC), recollection (y-intercept), and familiarity (d′) for items in each Object Condition for Experiments 1a and 1b

				DPSD Model Parameters		
Exp.	Object Condition	Hit Rate	False Alarm	*AUC*	*y-intercept*	*d′*
1a	Audiovisual Congruent	73.33 (13.50)	18.22 (10.27)	0.80 (0.09)	0.46 (0.22)	0.79 (0.48)
	Visual Neighbor	70.80 (12.43)	18.22 (10.27)	0.80 (0.09)	0.41 (0.20)	0.84 (0.57)
	Audiovisual Control	69.67 (12.29)	18.22 (10.27)	0.79 (0.08)	0.36 (0.21)	0.84 (0.61)
1b	Audiovisual Congruent	75.53 (13.84)	17.42 (13.98)	0.82 (0.12)	0.39 (0.27)	1.11 (0.78)
	Visual Neighbor	73.73 (14.02)	17.42 (13.98)	0.81 (0.12)	0.39 (0.27)	1.06 (0.74)
	Audiovisual Control	74.60 (15.24)	17.42 (13.98)	0.82 (0.12)	0.35 (0.28)	1.16 (0.84)

Standard deviations are shown in parentheses.

**ROC analysis.** Experiments 1a and 1b: For the ROC analysis, we calculated the cumulative hit rates (the proportion of old items correctly identified as old) for items in each Object Condition at each response criterion, and calculated the false-alarm rates (the proportion of new items incorrectly identified as old) at each response criterion to analyze the underlying ROC (Yonelinas & Parks, 2007). Each subsequent point on an ROC curve relates the hit and false-alarm rates as participants increasingly relax their criteria for classifying an item as *old*, from “definitely old” to “definitely new.” In line with the dual-process signal detection (DPSD) analysis of ROCs, the leftmost point includes both “recollect” and “definitely old” responses (Yonelinas et al., 2010). While the remember-know procedure relies on subjective reports of recollection- versus familiarity-based recognition to dissociate these processes, the DPSD model instead estimates these processes based on hit rates and false-alarm rates at each level of confidence through ROCs. The benefit of this ROC method is that it does not require participants to accurately introspect and assess the source of their own memory. However, we included the “recollect” response option to allow us to conduct remember-know analyses to assess whether our results converge across different commonly-used process-dissociation methods. We have included remember/know analyses for both Experiments 1a and 1b in the Supplemental Materials, and we note here that all results converge with the ROC results, except for the specific case noted in the Experiment 1 Discussion and Supplement (Appendix A).

**Fig. 2:**
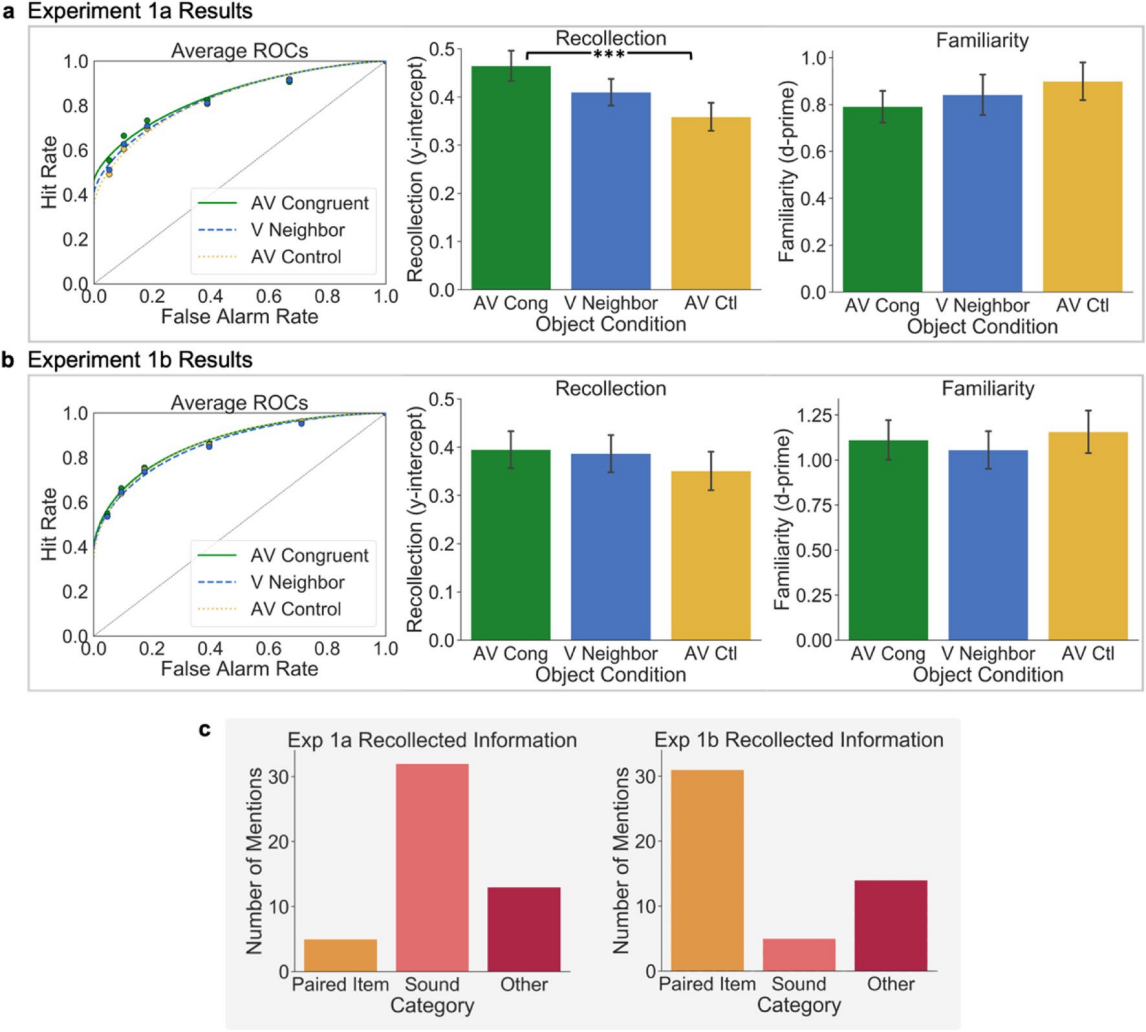
Results for Experiments 1a and 1b. **a.** The left graph depicts the averaged observed ROCs (points) for each Object Condition from Experiment 1a and corresponding Dual-Process Signal Detection (DPSD) functions. The center and right graphs show mean *y-intercepts* and *d-prime* as DPSD-based metrics of recollection and familiarity, respectively. The center graph shows recollection is greater for items encoded in the Audiovisual Congruent (AV Cong) condition than the Audiovisual Control (AV Ctl) condition. **b.** The left graph depicts ROCs for Experiment 1b, and the center and right graphs show mean *y-intercepts* and *d-prime* as DPSD-based metrics of recollection and familiarity, respectively. **c.** Mentions of each type of recollected information from the debriefing questionnaire, including mentions of the item the object was paired with during encoding (Paired Item), the sound played at encoding (Sound), or any other information about the task or objects (Other). All error bars illustrate standard error of the mean. (Color figure online)

For statistical analyses, individual ROCs were constructed for each participant at each level of Object Condition, and the points in the ROC graph of Fig. 2a reflect the average observed hit and false-alarm rates for these conditions across participants. The same false-alarm rates for new items were used across the three Object Conditions, which allows us to identify each participants’ individual criteria for judging an item as new across confidence levels to compare with hit rates at the same confidence levels. DPSD models were fit to each participants’ ROCs, and the average ROC model for each group is shown in Fig. 2a. To compare overall differences in memory performance between conditions, AUC was calculated using the trapezoidal rule for each participant’s observed ROCs in each Object Condition and compared via one-way RM ANOVA and Bonferroni corrected post hoc pairwise *t* tests. Parameter estimates derived from DPSD model-based ROCs were used to compare two constructs of interest from the dual-process model of recognition memory, namely the *y-intercept*, which estimates recollection, and *d-prime* (*d′*), which estimates familiarity. In the DPSD model, the *y-intercept* estimates the hit rate when the false alarm rate is equal to 0, making it a threshold measure of memory that represents recollection. Model-derived *d′* measures hit rates relative to false-alarm rates across the entirety of the curve, which quantifies the contribution of familiarity. These estimates were also compared via individual one-way RM ANOVAs and Bonferroni adjusted post hoc pairwise *t* tests.

Finally, to assess whether encoding task performance is related to recognition memory performance, we ran exploratory correlation analyses for both experiments. For both experiments, encoding task performance was assessed as percent correct, and this was related to hit rate on the recognition tasks.

##### **Debriefing questionnaire analysis**

Experiments 1a and 1b: To perform an exploratory assessment of the types of details that were retrieved about objects on trials for which participants respond with “recollect,” we coded the open-ended responses to the debriefing survey for mentions of specific items and features recollected from the encoding task (Fig. 2c). All responses included mention of an item from the encoding task along with a feature that was recollected. Responses that referred to objects and their accompanying sound were labeled as “Sound” recollections (e.g., “I remembered the dog because it was shown along with a ‘bark’ sound”). Responses that referred to the item that an object was paired with at encoding were labeled as “Paired Item” (e.g., “I remembered the toaster because it was paired with the tea kettle”). Other recollected aspects of the encoding experience related to the task or personal opinions or thoughts were labeled as “Other” recollections (e.g., “I remembered the elephant because elephants are my mom’s favorite animal”).

### Results

#### ROC analysis

##### **Recollection and familiarity**

ROC analyses were conducted in order to determine if recollection or familiarity for objects differed as a function of their occurrence with a congruent sound, appearance in the context of the other object with a congruent sound, or in the presence of white noise.

Experiment 1a: A one-way repeated measures analysis of variance (RM ANOVA) showed a significant effect of Object Condition on recollection (*y-intercept*), *F*(2, 98) = 6.55, *p* = .002, η_p_^2^ = 0.12 (Fig. 2), with higher *y-intercepts* for items in the Audiovisual Congruent condition than in the Audiovisual Control condition, *t*(49) = 4.41, *p* = .0002, Cohen’s *d* = 0.49; there was no significant difference between items in the Audiovisual Congruent and Neighboring Visual conditions, *t*(49) = 1.71, *p* = .28, Cohen’s* d* = 0.26, or the Audiovisual Control and Neighboring Visual conditions,* t*(49) = −1.64, *p* = 0.32, Cohen’s* d* = −0.25. Bayes factors provided very strong evidence for the difference between y-intercepts for the Audiovisual Congruent and Audiovisual Control conditions (*BF*_10_ = 386.31), and only anecdotal evidence for the null hypothesis for differences between y-intercepts for the Audiovisual Congruent and Neighboring Visual conditions (*BF*_01_ = 1.67) and Audiovisual Control and Neighboring Visual conditions (*BF*_01_ = 1.85). A one-way RM ANOVA did not show a significant effect of Object Condition on familiarity (*d′*), *F*(2, 98) = 0.66, *p* = 0.52, η_p_^2^ = 0.01 (Fig. 2a). These results indicate that recollection was higher for the audiovisual congruent object than the other objects, replicating our previous results. However, there was no memory benefit for the Neighboring Visual object that co-occurred with the Audiovisual Congruent object, suggesting that the benefit conferred by the congruent multisensory event did not transfer to other objects in the same visual context.

Experiment 1b: A one-way RM ANOVA showed no significant effect of Object Condition on recollection (*y-intercept*), *F*(2, 98) = 0.59, *p* = .56, η_p_^2^ = 0.012, or familiarity (*d′*), *F*(2, 98) = 0.51, *p* = .60, η_p_^2^ = 0.010. (Fig. 2b). These results suggest that the auditory stimulus had no effect on visual memory when the encoding task explicitly required a relational comparison of the two visual objects.

#### AUC

Experiment 1a: A one-way RM ANOVA did not show a significant effect of Object Condition on general recognition memory (AUC) *F*(2, 98) = 0.65, *p* = .53, η_p_^2^ = 0.01. This suggests that the auditory stimulus did not impact recognition memory accuracy beyond recollection-based recognition, which is unsurprising given that familiarity was also not significantly impacted.

Experiment 1b: A one-way RM ANOVA did not show a significant effect of Object Condition on general recognition memory (AUC) *F*(2, 98) = 1.06, *p* = .35, η_p_^2^ = 0.021. This suggests that the sound stimuli did not have an impact on overall recognition memory.

#### Encoding and memory performance relationship

For both experiments, we explored whether there were associations between successful encoding task performance and subsequent recognition.

Experiment 1a: The correlation was not significant between performance on the encoding task (*M* = 60.27%, *SD* = 5.60%) and the hit rate on the recognition task across participants (*M* = 76.52%, *SD =* 6.76%), *r*(50) = .009, *p* = .9504.

Experiment 1b: There was a significant positive correlation between performance on the encoding task (*M* = 75.93%, *SD =* 18.29%) and the hit rate on the recognition task (*M* = 78.60%, *SD* = 10.75%), *r*(50) = .41, *p* = .003.

#### Debriefing questionnaire

We analyzed participants' responses to the open-ended question in the debriefing questionnaire, which asked them to describe the features of objects they relied on to provide their recollection responses.

Experiment 1a: Out of a total of 50 responses, five (10%) mentioned the Paired Item, 32 (64%) mentioned the Sound, and 13 (26%) mentioned some Other aspect of the encoded event (Fig. 2c). This suggests that out of the features present at encoding that could have been recollected (e.g., the paired item, the sound, task-related information), the sounds matching Audiovisual Congruent stimuli were recollected and stood out compared with other features of the encoded event.

Experiment 1b: Out of a total of 50 responses, 31 (62%) mentioned the Paired Item, five (10%) mentioned the Sound, and 14 (28%) mentioned some Other aspect of the encoded event (Fig. 2c). This suggests that out of the features present at encoding that could have been recollected (e.g., the paired item, the sound, task-related information), participants were more likely to mention the Paired Item later on than the sound. This is in contrast to Experiment 1a (Fig. 2c) which showed the opposite pattern for the Paired Item and Sound mentions.

### Experiment 1a and 1b discussion

The goal of these complementary experiments was to assess whether multisensory object processing supports memory for nearby visual objects present at encoding using two different encoding tasks. In the encoding tasks, participants made size judgements about one of the two objects presented on each trial (Experiment 1a) or rated their semantic relatedness (Experiment 1b), in tasks that were designed to assess the impacts of multisensory processing on memory for nearby objects under conditions that elicit more individual versus relational processing, respectively. The ROC analysis for Experiment 1a replicated our previous findings, indicating that recollection-based recognition memory was superior for objects paired with congruent sounds during encoding, while familiarity was not different between Object Conditions. However, results did not align with our main hypothesis that recollection would also be significantly better for Neighboring Visual objects than Audiovisual Control objects. Bayes factors suggest anecdotal evidence that this is the case, though this comparison did not reach statistical significance. The remember-know analysis (see Appendix A) did actually show that Visual Neighbor items were remembered (recollected) at significantly higher rates than the Audiovisual Control objects. Unlike the ROC analysis, the remember-know procedure relies on participants’ ability to accurately determine the source of their own memory. This provides some evidence of a memory benefit for visual objects encoded within the context of congruent audiovisual objects. Overall, results from Experiment 1a did not provide strong, converging evidence that the presence of audiovisual objects also supports individual memory for a neighboring visual item. However, they did replicate our previous findings showing improved memory for the congruent audiovisual object itself and that the recollection benefit did not come at a cost to memory for nearby visual objects.

Experiment 1b was motivated by the possibility that the retrocue task in Experiment 1a emphasized processing each item alone, and that this may have led objects to be processed *more* individually than they would be in a naturalistic setting where they might be found co-occurring. However, results from Experiment 1b did not align with our initial hypotheses or with results of Experiment 1a. When the encoding task emphasized the associative relations between objects, the Audiovisual Congruent objects were no longer better recollected than the Audiovisual Control or Neighboring Visual objects. This may be because elaboration on the meaning and relation between the two objects improved memory over-and-above the memory benefits of audiovisual encoding. This idea is supported by the overall higher hit rates and lower false-alarm rates compared with Experiment 1a (see Table 1). Further, in Experiment 1a, Debriefing survey responses showed that participants most often mentioned congruent sounds when making recollection judgements rather than the paired item or some other detail from the encoding task, whereas in Experiment 1b, there were many more mentions of the paired objects than sounds. This could mean that the sounds were the most notable features aiding recollection in Experiment 1a, whereas the paired items drove recollection more in Experiment 1b. While the lack of effect of congruent audiovisual object processing on memory was unexpected, this provides new insight on the conditions under which audiovisual processing does not facilitate memory. However, more research is necessary to fully understand these boundary conditions in the multisensory benefit.

Contrary to expectations, neither Experiment provided strong evidence that the audiovisual recollection benefit for a single object extends to nearby unisensory objects. One reason for this may be that sounds only enhance encoding of the object producing the sound and do not extend to multi-object memories. Another reason, however, may be that our recognition memory task only measured memory for single objects and not for the association between the objects. The multisensory event may have generated a benefit for associative memory between the two objects, but our memory test was not sensitive to benefits in associative learning given that we tested memory for objects individually. This idea motivated Experiment 2, in which we test whether encoding audiovisual objects improves associative source memory for the context in which it was encoded.

## Experiment 2

**Fig. 3:**
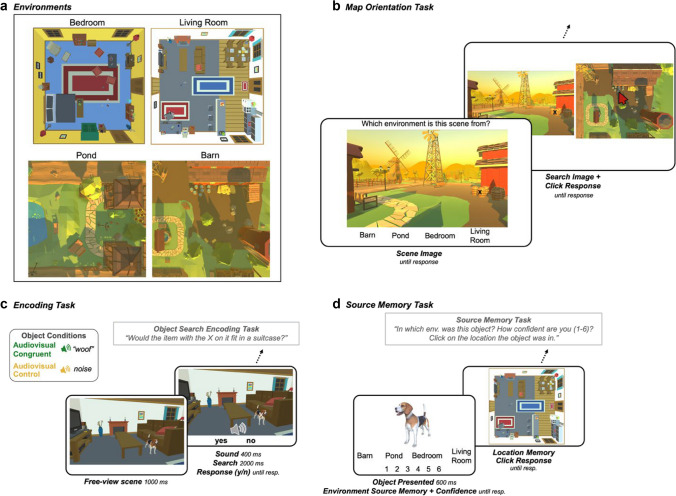
Experiment 2 design. a. Overhead view of the four environments used in this experiment, two indoor house environments, and two outdoor farm environments. b. Map Orientation Task in which participants identify which environment a scene image is from, then search for a target “x” in the scene and click on the location on the map where the “x” is located. c. Object Search Encoding Task in which participants search for the object with an “x” on it and make a size judgement about that object. d. Source Memory Task in which participants indicate in which environment they encoded the object (Environment Source Memory), confidence (1–6) in that judgement (Confidence), and then click on the location on the map where the object was encoded (Location Memory). (Color figure online)

The goal of this experiment was to test the hypothesis that multisensory object encoding facilitates memory for the context in which an object is embedded. Unlike the previous experiments, the context was defined by visual scenes within which the target objects were embedded. This approach emulates more naturalistic associations between objects and their context. To examine memory for the context, we assessed source memory rather than individual item recognition memory. Participants first familiarized themselves with four virtual environments: a barn area, a pond area, a living room, and a bedroom, learning their layouts and the objects in them (Fig. 3a–b). They then completed an audiovisual encoding task, in which they were presented with target objects embedded within scenes from these environments, along with a sound that was congruent to the target object, or a control sound (white noise). Participants searched for the target object (demarcated with an *X*) and made a size judgement about that object (Fig. 3c). In a surprise source memory test, participants indicated which of the four environments the item had been encoded in, their confidence in that memory, and, on a map of that environment, clicked on where the object had been located (Fig. 3d).

### Method

#### Participants

A total of 209 students from the University of California, Davis, participated in exchange for partial course credit, and 68 students were included in analyses (53 identified as female, 12 identified as male, and three identified as nonbinary, *M*_age_ = 19.40 years; see Supplement Table 6 for the full sample demographics). Our preregistered exclusion criteria were based on performance (less than chance level performance on either the encoding [*n* = 6] or memory tasks [*n* = 1]), use of the entire range of confidence options on the memory test (*n* = 112), and based on responses to the debriefing survey. From the debriefing survey, participants were excluded if they participated in a somewhat or very noisy testing environment, did not have consistent audio available during the study (*n* = 17), exerted no effort or little effort in completing the study (*n* = 5), or they reported that they did not understand any of the tasks (*n* = 0). An a-priori power analysis for one-tailed *t* tests determined that we would need a sample size of 34, and as with Experiments 1a and 1b, we doubled this for our target sample of 68. This sample size was preregistered, and data were collected until we reached 68 participants postexclusion. Participants were ineligible to participate in Experiment 2 if they had participated in Experiments 1a or 1b.

#### Materials

Materials for this experiment included most of the same objects as in the previous experiments, embedded within scenes from virtual environments, and deviations are described below.

##### **Environments**

Four environments were obtained from the Unity 3D Asset store (https://assetstore.unity.com/3d). These 3D environments consisted of two house environments (Bedroom & Living Room) and two farm environments (Barn & Pond; Fig. 3a). These environments were chosen because the majority of the objects used in the previous experiments reasonably fit within either the farm or house environments. Further, having an indoor set of environments and an outdoor set of environments allowed us to counterbalance the objects across two environments per object to control for memory biases based on the memorability of features of a specific environment, rather than the memorability of objects based on the Object Condition. The two farm environments were each approximately 7 × 7 meters, and the house environments were each approximately 5 × 5 meters. The environments were each filled with items typical to that space (e.g., the barn area has hay bales, a wheelbarrow, barrels). The overhead view of the map of each environment was split into a 5 × 5 grid of possible locations for objects within the encoding task. Of the 25 possible locations per environment, three were removed from each because they did not contain a “valid” placement for objects (e.g., in the pond). This grid structure was implemented to ensure that visual objects were evenly distributed across the environments. Each cell was edited to include at least one object in order to provide nearby contextual items for each encoded object. For the *Environment Orientation Task* (described below), point-of-view images of the environments were taken from ground level in various areas around the map without additional experimental objects added in. Each map also had map images taken from above, which were used in the map orientation task and the memory test.

##### **Objects**

We used 88 visual objects, which were divided into two groups, with 44 corresponding to the farm environments, and the other 44 corresponding to the house environments (see Supplemental Materials for full lists of items). The majority of these objects were the same as those used in Experiments 1a and 1b, though in order to more closely match each environment, six objects were replaced with four new ones which better matched the chosen environments. Additionally, some items (e.g., the leopard) were now used as *toys* within the environments rather than as the real version of the objects, which further served to improve the correspondence of objects and their environments. New visual objects also came from the Unity 3D asset store, and new sounds that were added came from the Multimost Stimulus Set. In previous studies, we presented objects in grayscale, though here, objects and scenes were presented in color in order to increase the realism of the environments and objects. During the encoding phase, objects were presented within the environments in scenes (see below for details), and during the memory test, objects were shown on their own, edited to fit within 500 × 500 pixels. Sounds used in this experiment were either congruent to the object or a control white noise sound, which were the same control sounds used in the previous experiments.

##### **Scenes**

Scenes were constructed by placing objects in one of the grid cells within the 5 × 5 map of locations excluding unrealistic locations for placing our objects (e.g., the pond). This left 22 locations for target object per environment. The objects in each cell were randomized so that performance in the memory phase could not be based on guessing related to typical locations of items. Therefore, some objects were in atypical locations within the environments (e.g., a hammer on a bench), but none were so out of place that they would be considered impossible. For each scene image, objects were placed anywhere within their designated cell, and screen captures were taken with the objects at ground-level and within the central-third of the image. The objects were a variety of sizes to emulate naturalistic size variation. Each object only appeared once, and was not visible in the background of other images, though the rest of the background images for each environment was consistent across the scenes. Each object was counterbalanced across Object Conditions and its two corresponding Environments, such that each scene could be paired with a congruent sound or with a control sound, and each object appeared in both environments once between subjects. For example, across subjects, the sheep appeared in both the barn area and in the pond area. For the target search phase of each trial, black or white *X*s were placed on the target object (depending on which was more easily visible) on the target object. Each image was presented at 1,000 × 1,000 pixels.

#### Procedure

As with the previous experiments, this experiment began with sample beeps for participants to use for sound level calibration, and all stimuli were presented online via personal computers through the online stimulus presentation software Testable (https://www.testable.org/). Participants completed the *Map Familiarization Phase*, then the *Map Orientation Task*, followed by the *Object Search Encoding Task*, then the surprise *Source Memory Task*, and finally the *Debriefing Questionnaire*. Time-to-complete the experiment was not recorded for each participant, but the experiment took approximately 30 minutes to complete during internal pilot testing.

##### **Map familiarization phase**

This block of stimuli was used to introduce participants to the layout of each environment and information within them. The goal of this was to encourage participants to consider features of the environmental context during the encoding task, even though there were no explicit instructions to do so during that task. In this block, participants were shown successive images of each environment, including an overhead map of the environment and four images of each environment from the first-person perspective (see Supplemental Materials Fig. 2 for examples). They were told to acquaint themselves with the environments because they would be asked questions about them in the next task. Participants were first shown the map and a broad view of an environment along with a short description of what the environment contains (e.g., “This is the Barn. It contains objects like a barn, a well, hay, and barrels. Have a look around”). They were then shown three additional pictures of each environment from a first-person perspective, followed by all five images together. This was done for each of the four environments, and the order of the environments was randomized for each participant. Each trial was displayed for five seconds before allowing participants to advance at their own pace.

##### **Map orientation task**

The purpose of this self-paced task was for participants to practice identifying which environment a given scene came from, and matching the first-person perspective scenes to their locations on the overhead map of the environments in order to complete the memory task later on. On each trial, participants viewed a scene from one of the four environments and indicated which environment it came from by clicking an on-screen button with the environment labels (Fig. 3b). They were then shown the image next to the map of the correct environment. On these trials, a small target (an *X*) was added to an object or location on the scene, and participants were asked to click on the overhead map the location that the target was shown in the scene. There were six scenes randomly presented from each environment, for a total of 24 environment identification trials, and 24 localization trials, and 48 trials in this block overall.

##### **Object search encoding task**

The encoding task consisted of 88 trials of a visual search and size judgement task within scenes from the environments. On each trial, participants viewed a scene from one of the environments for one second before the onset of a target *X* on top of the target object and a sound. The sound corresponded to one of the two Object Conditions, such that it was congruent with the target object (Audiovisual Congruent), or it was composed of white noise (Audiovisual Control; Fig. 3c). After the onset of the target, the image disappeared after 2,000 ms, and participants were asked to indicate whether the item with the target *X* on it would fit in a standard suitcase. Once participants responded, there was a 600-ms intertrial interval. Scenes from each environment were presented randomly. Across versions of the experiment, the Object Conditions were counterbalanced, such that every object was paired with a congruent sound and a control sound in one version of the experiment. Additionally, each target item was counterbalanced across its two congruent environments (e.g., the stapler was shown in both the living room and bedroom). This counterbalancing scheme resulted in eight versions of the experiment, to which participants were pseudorandomly assigned by Testable to result in an even distribution of experiment versions completed across participants. This task was designed to encourage participants to process the scenes prior to searching for and making a size judgment about the target object which was either an audiovisual or control object. This allowed us to later assess the impact of the Object Conditions on memory for the target objects and the contexts in which they appeared.

##### **Source memory test**

The surprise source memory test included 88 two-part trials to test memory for the environments in which each object was encoded (Environment Source Memory), confidence in that judgment (Confidence), and memory for the precise location within the environment that the item was originally placed (Location Memory). Environment Source Memory: On each self-paced trial, participants saw a target object from the encoding task and indicated which of the four environments, “barn,” “pond,” “bedroom,” and “living room,” the object was originally located in (Fig. 3c). Confidence: Participants indicated their confidence in their Source Memory judgement on a scale from 1 (*not confident at all*) to 6 (*extremely confident*). Location Memory: Participants were shown a map of the environment that they selected, and asked to use their mouse or trackpad to click on the location within the map where the object originally appeared. If the participant selected the wrong environment during the first part of the trial, they were taken to the incorrect map, and only the Environment Source Memory of these trials were analyzed. This task was designed to test whether Object Condition influenced memory for the context in which an object was encoded, confidence in that memory, and precision of the visuospatial memory for that item within the environment. Participants were instructed to use the entire range of response options across trials.

##### **Debriefing questionnaire**

As with our previous experiments, we had participants complete a debriefing survey after the end of the experiment. The survey was similar to previous experiments, with the addition of questions specific to this study, such as whether participants expected a memory test, whether there were any individual tasks of the study in which they put in less effort, and an introspective question asking for participants to give an example of what they believe helped them remember the items that they did remember (see Supplemental Materials for a full list of questions).

#### Data analysis

Our pre-registered analyses included comparisons between source memory performance for items paired with congruent sounds and control sounds during the Object Search Encoding Task. We deviated from some aspects of the pre-registered analysis plan to opt for a more appropriate analysis for our data, as described below, though the hypotheses tested remain the same. The raw data and results from pre-registered analyses can be found in the experiment folder on the OSF page (https://osf.io/sep3r/) along with the analyses presented in the paper. Results from the Map Orientation Task were used for comparison with Location Memory and descriptive statistics for that task are reported in the Location Memory section of the results.

#### Source memory

##### **Environment source memory**

To assess source memory for the environment in which objects were encoded across Object Conditions, we performed a* t* test comparing accuracy (*percent correct*) between the Audiovisual Congruent and Audiovisual Control Object Conditions. We compared percentage correct rather than *d*′ because percentage correct is a more common measure for analysis of *m*-AFC tasks and because both of these measures convey relative performance accurately within forced-choice tasks (Brady et al., 2021; Stanislaw & Todorov, 1999).

##### **Confidence**

As an additional measure of memory strength, we compared confidence ratings between conditions. To do this, we took the average confidence rating (1–6) for each participant in each condition for correct responses (hits), and compared these averages between conditions using a *t* test.

Although our preregistered analysis plan included an ROC analysis based on confidence responses, hit rates, and false-alarm rates, we ultimately decided the number of items that we had per condition would not be enough to properly characterize ROC functions. Yonelinas and Parks (2007) state that ROCs constructed from fewer than 50 responses per condition (i.e., 50 old items and 50 new items) can be noisy and irregularly shaped, impairing the ability to properly characterize the function. Whereas our Experiments 1a and 1b had 30 items per condition and 90 new items (total of 180 items), Experiment 2 only had 45 items per condition, all of which had been seen before. We deemed this to be too few trials to reliably carry out the ROC analysis. However, because confidence ratings have been shown to track with accuracy and memory strength (Brady et al., 2021; Mickes et al., 2011), we have included the confidence data here using the alternative analysis plan.

##### **Location memory**

Accuracy (Euclidean distance) was compared between Object Conditions using a paired-samples *t* test. Trials with incorrect Environment Source Memory were excluded from this analysis.

#### Encoding and memory performance relationship

To assess whether relationships exist between encoding task performance and recognition memory performance, we ran an exploratory Pearson correlation analysis between percentage correct on the size judgement encoding task, compared with percentage correct on the environment judgement of the memory test.

#### Debriefing questionnaire

**Fig. 4:**
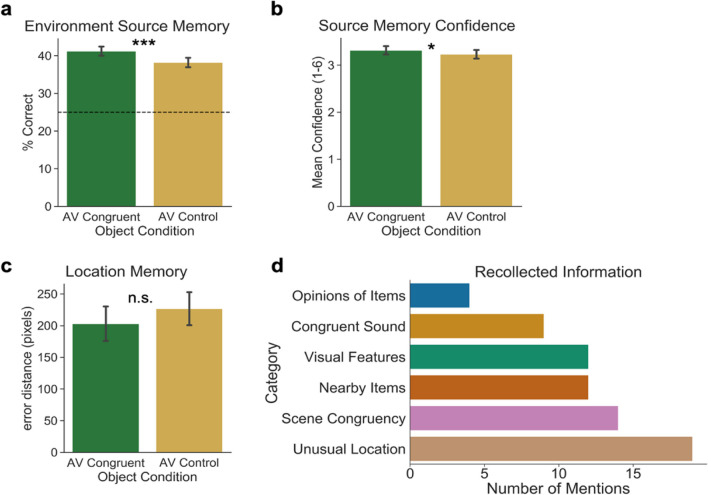
Experiment 2 results. a. Average accuracy (% correct) for environmental context source memory between Object Conditions. Accuracy was significantly higher in the Audiovisual Congruent condition than the Audiovisual Control condition. b. Source memory confidence between Object Conditions for correct environmental context source memory. Confidence was significantly higher for items in the Audiovisual Congruent condition than those in the Audiovisual Control condition. c. Error of Location Memory for where the object was located in the scene. d. Mentions of information that was reported to facilitate memory for the environment the object was encoded in as reported in the debriefing questionnaire. Error bars illustrate standard error of the mean

We performed an exploratory analysis of responses to the open-ended debriefing survey prompt asking participants to report an example of something they believed helped them remember the environment that objects were located in during encoding. For this analysis, we coded responses for mentions of different types of information (e.g., mentions of memory for sounds, visual features, or background scene objects) to assess themes across participants. Responses that included mentions of multiple types of information were counted in each of those categories, so the total number of responses exceeds the number of participants. We report counts of mentions of information from the emergent categories in Fig. 4.

### Results

#### Source memory

##### **Environment source memory**

For this analysis, we assessed memory for the environment in which each item was encoded across the two Object Conditions. A one-tailed, paired-samples *t* test revealed a significant effect of Object Condition on Environment Source Memory (percentage correct), *t*(67) = 2.47, *p* = .008, Cohen’s *d* = 0.30, such that memory performance was better for objects encoded in the Audiovisual Congruent condition (*M* = 0.41, *SD* = 0.10) than for objects encoded in the Audiovisual Control condition (*M* = 0.38, *SD* = 0.10) (Fig. 4a). Additionally, the Bayes factor suggests moderate evidence for this finding (*BF*_10_ = 4.47). Our preregistered *t* test comparing *d′* rather than percentage correct between the two Object Conditions yielded an effect with almost identical statistics, which are reported in the Supplemental Materials on OSF (https://osf.io/sep3r/).

**Fig. 5:**
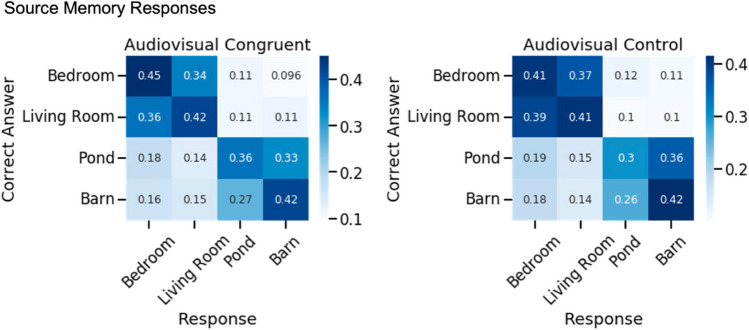
Experiment 2 Source Memory Response Matrices. These matrix heatmaps illustrate the proportions of source memory responses in each environment, with the correct environments on the *y*-axes, and the responses on the *x*-axes for objects encoded in the Audiovisual Congruent and Audiovisual Control conditions, with the correct responses on the diagonal, and false alarms on the off-diagonals. The darker blue diagonal for objects encoded in the Audiovisual Congruent condition (left) illustrates the more precise memory for where these objects were encoded, relative to objects encoded in the Audiovisual Control condition

To visualize performance across the environments, we plotted a confusion matrix with the true encoding environments and the response environments (Fig. 5). False alarms most often went to the environment that was most similar to the original environment. For example source memory was most often confused between the two indoor house environments and the two farm environments, which is illustrated by the darker boxes in Fig. 5. We therefore conducted an exploratory analysis to test the hypothesis that congruent sounds improved general source memory for the environment category (i.e., the object was encoded in an indoor [house] or outdoor [farm] environment). An exploratory, one-tailed, paired-samples *t* test showed no significant effect of Object Condition on indoor/outdoor Source Memory (percentage correct), *t*(67) = 0.73, *p* = .77, Cohen’s *d* = 0.09, *BF*_01_ = 2.92. The contrast between this test and the significant difference in specific Environment Source Memory reported above suggests that congruent sounds improved memory for the specific environment in which an object was encoded rather than a more general memory for whether an object was encoded in an indoor or outdoor environment, and the Bayes factor provides anecdotal evidence for this null hypothesis.

##### **Confidence**

To assess whether sound congruence affected memory confidence, we analyzed average confidence ratings in memory across Object Conditions. A one-tailed, paired-samples *t* test on confidence ratings showed a significant effect of Object Condition on memory Confidence, *t*(67) = 1.66, *p* = .050, Cohen’s* d* = 0.113, such that confidence was higher for objects paired with congruent sounds at encoding (*M* = 3.32, *SD* = 0.72) than for objects paired with control sounds at encoding (*M* = 3.23, *SD* = 0.76). The Bayes factor provides anecdotal evidence for the null hypothesis for this effect (*BF*_01_ = 0.98; Fig. 4b).

##### **Location memory**

A one-tailed, paired-samples *t* test on memory for the specific object in the Location Judgement (*Euclidean distance in pixels*) did not show a significant effect of Object Condition, *t*(67) = 0.64, *p* = .26, Cohen’s* d* = 0.06; the distance between the true object location and the reported location was not different for objects encoded in the Audiovisual Congruent condition (*M* = 203.12, *SD* = 187.10) than for objects encoded in the Audiovisual Control condition (*M* = 226.81, *SD* = 184.18) (Fig. 4c). While performance between these two conditions was not significant, this pattern of results is consistent with our hypothesis that memory for object location would be more precise for objects encoded with a congruent sound than a meaningless control sound. The Bayes factor suggests only anecdotal evidence for the null hypothesis between these groups (*BF*_01_ = 3.03). Results from the Map Orientation Task showed overall higher performance (*M* =118.07, *SD* = 105.72) than on Location Memory, suggesting that they were able to perform the translation from scene-view to map-view more accurately when they were looking at both the scene and map concurrently. This suggests that performance was low for Location Memory not because participants could not perform the task properly, but because the task was more difficult and/or Location Memory was not very precise.

#### Encoding and memory performance relationship

We used a correlation analysis to explore the relationship between successful encoding task performance and subsequent recognition memory. There was not a significant correlation between encoding task performance (*M* = 81.42%, *SD* = 9.68%) and recognition task performance (*M* = 39.71%, *SD* = 8.83%), *r*(68) = .18, *p* = .1127.

#### Debriefing questionnaire

For this analysis, we assessed responses to the open-ended debriefing questionnaire prompt which asked participants to report an example of something that helped them remember the environment that objects were located in during encoding. Four participants did not provide responses to the prompt, and three participants provided two responses. All other participants provided one response, resulting in 70 responses that were used in the analysis. Of the 70 responses, six categories of information emerged, including (1) personal opinions of or connections to items (5.71% of responses); (2) memory for object-congruent sounds (12.86% of responses); (3) visual features of objects, such as size, color, or shape (17.14% of responses); (4) memory for items nearby the target objects (17.14% of responses); (5) congruency of an object to a scene (20% of responses); and (6) memory that an object was placed in an unusual or unexpected location (27.14% of responses; Fig. 4d). The proportions of mentions indicate that the most salient scene features driving memory for the environment that an object was encoded in were related to how well an object fit in the environment or how surprising it was to see the item in the location that it was in. This is unsurprising given that object-scene incongruency is known to capture attention (e.g., Biederman, 1972; Henderson et al., 1999). Interestingly, nine participants (12.86%) reported that congruent sounds helped them remember where objects were located within the environment. One participant reported, “When the sound matched, like the bell in the barn, I was able to recall the location almost instantly.” While sound congruency was not the most frequently reported piece of memory-supporting information, the presence of these mentions supports our hypothesis that encoding a visual object along with a congruent sound can lead to better memory not only for the object itself and the sound, but also other features of the encoding context.

### Experiment 2 discussion

The results from Experiment 2 supported our hypothesis that encoding an object along with a congruent sound facilitates source memory for features of the object’s encoding context. This was demonstrated in the comparison of performance on the 4-AFC Environment Source Memory for items encoded with congruent sounds and those with meaningless control sounds. This finding is particularly striking given the task did not require attention to the sounds or the environment, and participants were unaware their memory would be tested. Self-reported confidence ratings showed that participants were also more confident in correct responses for objects in the Audiovisual Congruent condition compared with objects in the Audiovisual Control condition. Further, we found that congruent sounds supported memory for the specific environment that the object was encoded in, rather than a more general sense of whether an object was encoded in an indoor or outdoor environment. Together, these results suggest that source memories for audiovisual objects are stronger and more precise than those for visual objects with no congruent sound information.

While we expected location judgements to be more precise for objects encoded in the Audiovisual Congruent condition, we did not find that to be the case, though performance was numerically better (smaller distance) than in the Audiovisual Control condition. We expect that this was at least in part due to very low performance on the location task, suggesting that participants made a large number of guesses. This is supported by debriefing survey responses, for which 85.29% of participants reported finding this task “very difficult,” and the remaining 14.71% reported finding the task “difficult.”

Overall, results were consistent with our main hypothesis, suggesting that hearing a sound when encoding a matching visual object can lead to better memory not only for the object itself, but also for its association with the environmental context in which it was encoded.

## General discussion

The goal of the present research was to investigate how multisensory object processing affects episodic memory for other object and context information present during encoding. In Experiment 1a, we found that pairing an object with a congruent sound yielded recollection benefits, without significantly improving or impairing memory for neighboring visual objects. Experiment 1b showed that increasing the task-relevance of the relation between an Audiovisual Congruent and a Neighboring Visual object during encoding eliminated the benefit of multisensory encoding altogether suggesting that relational encoding overpowers and effectively negates the advantage typically gained from processing a multisensory object. Lastly, Experiment 2 showed that memory for the environment in which an object was encoded was better for those paired with congruent sounds than meaningless control sounds. Together, results from these studies provide evidence that audiovisual object processing impacts episodic memory by strengthening memory for that object without disrupting memory for nearby objects and by improving memory for the scene context in which the object was encoded. These findings extend previous research on multisensory memory benefits and have implications for understanding episodic memory formation in naturalistic encoding contexts in which multisensory stimuli are pervasive.

The item-based memory test used in Experiment 1a allowed us to uncover an important aspect of the multisensory memory effect, which is that it does not negatively impact memory for nearby visual items. By testing memory for items individually, rather than memory for the association between co-presented objects, we found that the memory benefit of multisensory object processing does not come at a cost to memory for individual objects co-presented alongside them. Previous work has shown better memory for items that were selectively attended during encoding, and worse memory for contextual information that was not attended (e.g., Uncapher & Rugg, 2009). While selective attention may still play a role in audiovisual memory benefits, our results suggest that if it does, it uniquely does not come at the cost to memory for other nearby objects.

Results from Experiment 2 are particularly striking given that neither the sounds nor the scene contexts were relevant to the encoding task, yet the object-congruent sounds led to better memory for the scene context. While studies have been conducted to characterize the factors that improve associative and source memory in this way (see Mitchell & Johnson, 2009; Yonelinas et al., 2022), none, to our knowledge, have shown improved source memory when the to-be-associated information is task-irrelevant. This suggests that multisensory object processing may improve the ability to recognize objects *and* to differentiate between episodic memories for a particular object by improving memory for where that object was encoded. These results complement the results from Experiment 3 of Duarte et al. (2022), in which they found better source memory for sounds that were congruent with a simultaneously presented visual object (e.g., *ribbit +* frog compared with *ribbit* + dog). In that study, the sounds were not relevant to the encoding task. Murray et al. (2022) took the associative memory test a step further to test memory for faces paired with visually or audio-visually presented names, though the associations were still in reference to a single object (a person), rather than a multisensory object plus some additional context. Experiment 2 of the present research shows that this source memory benefit extends to information outside of the object itself to support memory for novel associations between congruent audiovisual objects and the context in which they appeared. Together, our results and those of previous studies characterize the multisensory memory effect as impacting memory for associations directly related to the constituent stimuli (i.e., the visual and auditory elements of a single object; Duarte et al., 2022), associations between faces and names (Murray et al., 2022), and incidentally encoded contextual scene information (the present study).

Proposed mechanisms underlying the multisensory memory effect are based largely on behavioral evidence for the robust memory representations created by audiovisual encoding. According to Dual Coding theory, audiovisual processing improves memory by building a memory trace with multiple paths to access that representation (Thompson & Paivio, 1994). They posited that auditory and visual codes of a single stimulus (i.e., a spoken and written word) are stored separately in memory and function as unique encodings of a single item, providing two independent memory codes that can be relied on for a single object. However, Murray et al. (2022) found that the audiovisual benefit for associative memory for faces and names was due to multisensory integration rather than simply having multiple independent memory traces, such that the benefit was greater when the audiovisual pairs were integrated into a single percept by comparing memory for synchronously versus asynchronously encoded audiovisual stimuli. This suggests that the creation of an integrated multisensory percept provides associative memory benefits over-and-above the benefits of just having a second redundant route to accessing the memory representation, and that the constituents of the audiovisual pair bind to the other information present at encoding as well. In their study, the audiovisual and contextual information were all associated with the same object (i.e., names, faces, and voices), but our study demonstrates how their multisensory encoding framework could be extended to show that the rich encoding of audiovisual objects benefits other objects as well.

While much of the previous research on multisensory memory has focused on individual objects and their corresponding sounds, there has been some work assessing the effects of sounds in memory for dynamic video clips. Some research has found a memory benefit for dynamic movie clips to be greater than static unisensory snapshots (Buratto et al., 2009; Matthews et al., 2007, 2010). Others have shown additional benefits of audiovisual dynamic rather than visual dynamic scenes (Meyerhoff & Huff, 2016; Meyerhoff et al., 2023). The study by Meyerhoff and Huff (2016) specifically found that audiovisual film clips were better remembered than unimodal visual or auditory clips, even when the auditory and visual stimuli are offset temporally, which they interpret as implying a distinct audiovisual integration process for scenes or dynamic information that relies on learned semantic associations more than low-level factors like temporal synchrony. This contrasts the findings of Murray et al. (2022), which did find a benefit of temporal synchrony between audiovisual stimuli at encoding on memory. This opens the possibility that there are distinct or multiple mechanisms by which audiovisual processing supports memory for individual objects and for episodic memories. Within the Meyerhoff and Huff study, and Experiment 2 of our study, it is possible that multisensory integration was not the main driver of the memory benefit, but rather the presentation of a sound that reinforced the visual identity. This could be addressed using a similar paradigm to our Experiment 2 with redundant unimodal information contrasted with redundant crossmodal information. Future research in this area will provide valuable insight into the potentially multifaceted impacts of multimodal information on memory for objects and events, which will be especially important for generalizing laboratory findings to the real world.

The benefit of multisensory object encoding on object-context binding and association building found in our study also has implications for understanding the mechanisms underlying episodic memory encoding in general. There is a great deal of evidence that memory for objects and object-context associations are behaviorally distinguishable and are supported by distinct neural mechanisms. For example, evidence from neuroimaging and patient studies suggests that the hippocampus plays an integral role in binding objects to their context to form the basis of episodic memories, while other areas of the medial temporal lobe, such as the perirhinal cortex, are involved in memory for individual objects (Davachi & Wagner, 2002; Diana et al., 2007; Ekstrom & Ranganath, 2018; Yonelinas et al., 2010, 2019). Duarte et al. (2022) found the multisensory memory effect to be specific to recollection-based recognition memory, with no effect on individual item familiarity, and results from the present study and Murray et al. (2022) provide evidence that multisensory processing at encoding facilitates the learning of novel associations. Given the role of the hippocampus in recollection, associative learning, and object-context binding, these studies suggest that the hippocampus may play a unique role in encoding multisensory objects. There is some evidence suggesting that the hippocampus is especially involved in creating novel associations across modalities (Borders et al., 2017; Kok & Turk-Browne, 2018). For example, Borders et al. (2017) had participants with hippocampal and broader MTL lesions study novel audiovisual and unisensory (visual–visual and audio–audio) stimulus pairs. Compared with healthy controls, both patient groups were significantly more impaired in their associative memory for audiovisual pairs, suggesting that the hippocampus is particularly necessary for building cross-modal associations. This lends credence to the hypothesis that the hippocampus plays a unique role in encoding audiovisual objects, though more research is necessary to understand how this region and other MTL regions contribute to audiovisual object-context binding. Studying these neural mechanisms will be important for understanding why multisensory object encoding impacts memory for context outside of the object itself, and will contribute to building ecologically valid models of episodic memory.

In summary, the present research provides novel evidence that encoding congruent audiovisual objects improves memory for both the audiovisual object and the environmental context in which it was encoded, and that the benefit does not come at a cost to memory for individual objects encoded nearby. This study extends previous work showing multisensory memory benefits to recognition memory (Duarte et al., 2022; Heikkilä et al., 2015, 2017; Lehmann & Murray, 2005; Matusz et al., 2017; Murray et al., 2022; Thelen & Murray, 2013; Thelen et al., 2015) to better our understanding of how multisensory processing impacts the formation of episodic memories. In more naturalistic settings, multiple mechanisms may work in tandem to produce unique memory outcomes for audiovisual objects and events. Because multisensory processing has also been shown to increase stimulus detection and attentional capture, these mechanisms may impact memory during real-world experiences. Understanding the interplay between these processes will ultimately help us uncover how memories are formed in real-world situations, in which visual objects and events are encoded in the context of other, multimodal information. Further, while other encoding strategies such as elaborative processing, increased attention, and mnemonic building have been shown to increase recollection or associative learning, they all require effortful shifts in controlled strategies. In contrast, our study demonstrates that the multisensory encoding results in memory benefits even under incidental encoding conditions. Further investigating the parameters of the multisensory memory effect and its underlying mechanisms will provide insights into memory systems and opportunities for the development of new strategies for improving memory and learning.

## Supplementary information

Below is the link to the electronic supplementary material.

Supplementary file1 (DOCX 670 KB)

## Data Availability

The raw data for all experiments are available at https://osf.io/sep3r/ and the design, hypotheses, and data analyses were preregistered for Experiments 1a and 2, with all exceptions and exploratory analyses documented as such within the manuscript. Trial files for each experiment defining stimulus presentations in Testable can also be found on the OSF page.

## Code availability

The code for analyses for all experiments are available at https://osf.io/sep3r/.
