# Supplementary material for: Multisensory processing impacts memory for objects and their sources
Source: Mem Cognit. Author manuscript; Available in PMC 2025 Mar 16. (PMC11868352; doi:10.3758/s13421-024-01592-x)
Supplement: Supplementary Materials [file NIHMS2057527-supplement-Supplementary_Materials.docx]

Supplemental Information for:

**Title: Multisensory processing impacts memory for objects and their sources**

**Authors:** Shea E. Duarte^1,2^, Andrew P. Yonelinas^1,3^, Simona Ghetti^1, 2^, & Joy J. Geng^1,2^

1. Department of Psychology, University of California, Davis, Davis, CA, 95618

2. Center for Mind and Brain, University of California, Davis, Davis, CA, 95618

3. Center for Neuroscience, University of California, Davis, Davis, CA, 95618

**Table 1.** Stimuli for Experiment 1a, sounds also used across experiments.

| **Old/New** | **Object Size** | **Object** | **Sound Description** |
| --- | --- | --- | --- |
| Old | Small | cup with straw | *Slurping from near-empty cup through straw* |
|  |  | snake | *Hiss* |
|  |  | toaster | *Toaster pop* |
|  |  | keyboard | *Keyboard typing* |
|  |  | spray bottle | *Spray mist* |
|  |  | bird | *Bird chirping* |
|  |  | light switch | *Click of switch turning on/off* |
|  |  | coins | *Coins chiming as they hit one another* |
|  |  | key | *Key jangling against one another* |
|  |  | tennis racket | *Racket hitting tennis ball* |
|  |  | camera | *Film camera shutter* |
|  |  | harmonica | *Note played on harmonica* |
|  |  | soda can | *Can being opened* |
|  |  | smartphone | *Phone ringtone* |
|  |  | xylophone | *Notes ascending on xylophone* |
|  |  | ping pong paddle | *Paddle hitting ball* |
|  |  | bowling pin | *Pins falling onto hardwood* |
|  |  | bat | *Bat hissing/chirp* |
|  |  | flute | *Note played on flute* |
|  |  | chick | *Baby chick chirping* |
|  |  | rat | *Rat squeaking* |
|  |  | clock | *Ticking clock* |
|  |  | matches | *Matchstick strike* |
|  |  | stapler | *Stapling paper* |
|  |  | cat | *Meow* |
|  |  | kettle | *Water boiling and bubbling* |
|  |  | blender | *Whirring* |
|  |  | hairdryer | *Air blowing* |
|  |  | tape | *Tape being cut from roll* |
|  |  | drill | *Electric drill whirring* |
|  |  | laptop | *Windows startup sound* |
|  |  | hammer | *Striking wood* |
|  |  | hen | *Chicken clucking* |
|  |  | scissors | *Snipping* |
|  |  | pencil | *Writing on paper* |
|  |  | teapot | *Teapot whistle* |
|  |  | cup | *Liquid slurped from mug* |
|  |  | book | *Pages turning* |
|  |  | wine bottle | *Cork popping out* |
|  |  | saw | *Sawing wood* |
|  |  | maracas | *Rattling* |
|  |  | basketball | *Basketball bouncing* |
|  |  | lighter | *Lighter clicking on* |
|  |  | frog | *Ribbit* |
|  |  | bell | *Ding* |
|  | Large | fireplace | *Fire crackling* |
|  |  | tiger | *Tiger roar* |
|  |  | washer | *Clothes tumbling and low engine whirring* |
|  |  | cymbal | *Cymbal crash* |
|  |  | axe | *Striking wood* |
|  |  | boat | *Boat horn* |
|  |  | jet | *Jet engine running* |
|  |  | helicopter | *Helicopter blades spinning and engine running* |
|  |  | drum | *Snare drum being hit* |
|  |  | printer | *Document printing* |
|  |  | guitar | *Notes strummed on guitar* |
|  |  | door | *Creaking open/closed* |
|  |  | leopard | *Leopard hiss* |
|  |  | goat | *Bleating* |
|  |  | crocodile | *Growl* |
|  |  | golfclub | *Striking golf ball* |
|  |  | car | *Car engine turning on* |
|  |  | horse | *Whinny* |
|  |  | billiards | *Billiards balls crashing into each other* |
|  |  | dog | *Bark* |
|  |  | cow | *Moo* |
|  |  | arcade game | *Video game beeps* |
|  |  | anvil | *Clank of hammer hitting anvil* |
|  |  | motorcycle | *Revving engine* |
|  |  | bear | *Bear roar* |
|  |  | sink | *Faucet running and turning off* |
|  |  | train | *Train running along tracks* |
|  |  | baseball bat | *Bat hitting baseball* |
|  |  | skateboard | *Skateboard rolling on concrete* |
|  |  | bicycle | *Bicycle bell* |
|  |  | toilet brush | *Scrubbing* |
|  |  | bow & arrow | *Release of arrow* |
|  |  | sled | *Sliding on snow* |
|  |  | chair | *Sliding along wood floor* |
|  |  | filing cabinet | *Sliding along tracks into closed position* |
|  |  | toilet | *Flushing* |
|  |  | microwave | *Beeping upon completion* |
|  |  | wolf | *Howl* |
|  |  | elephant | *Elephant wail* |
|  |  | penguin | *Penguin chirps* |
|  |  | deer | *Deer bleating* |
|  |  | pig | *Oinks* |
|  |  | piano | *Notes played on piano* |
|  |  | sword | *Sword unsheathing and hitting metal* |
|  |  | goose | *Goose honk* |
| New |  | wrench |  |
|  |  | whale |  |
|  |  | watering can |  |
|  |  | walkie talkie |  |
|  |  | scooter |  |
|  |  | shark |  |
|  |  | salamander |  |
|  |  | record player |  |
|  |  | rhino |  |
|  |  | rabbit |  |
|  |  | fridge |  |
|  |  | fish |  |
|  |  | crab |  |
|  |  | frypan |  |
|  |  | camel |  |
|  |  | butterfly |  |
|  |  | bus |  |
|  |  | radio |  |
|  |  | air hockey table |  |
|  |  | briefcase |  |
|  |  | calculator |  |
|  |  | candle |  |
|  |  | football |  |
|  |  | hoe |  |
|  |  | lamp |  |
|  |  | microphone |  |
|  |  | notepad |  |
|  |  | plant |  |
|  |  | pliers |  |
|  |  | scorpion |  |
|  |  | screw |  |
|  |  | screwdriver |  |
|  |  | snail |  |
|  |  | soccer ball |  |
|  |  | spider |  |
|  |  | spoon |  |
|  |  | table |  |
|  |  | turtle |  |
|  |  | umbrella |  |
|  |  | water bottle |  |
|  |  | whisk |  |
|  |  | eraser |  |
|  |  | octopus |  |
|  |  | pen |  |
|  |  | toucan |  |
|  |  | zebra |  |
|  |  | bed |  |
|  |  | cake stand |  |
|  |  | closet |  |
|  |  | couch |  |
|  |  | espresso maker |  |
|  |  | mokapot |  |
|  |  | stove |  |
|  |  | barrel |  |
|  |  | bucket |  |
|  |  | crowbar |  |
|  |  | rope |  |
|  |  | scale |  |
|  |  | tv |  |
|  |  | bedside table |  |
|  |  | mirror |  |
|  |  | tree |  |
|  |  | bathtub |  |
|  |  | spatula |  |
|  |  | basket |  |
|  |  | box |  |
|  |  | duffel |  |
|  |  | giftbox |  |
|  |  | pallet |  |
|  |  | shelf |  |
|  |  | suitcase |  |
|  |  | trash can |  |
|  |  | treasure chest |  |
|  |  | extinguisher |  |
|  |  | tire |  |
|  |  | chess |  |
|  |  | cone |  |
|  |  | streetlight |  |
|  |  | hydrant |  |
|  |  | ladder |  |
|  |  | mailbox |  |
|  |  | street sign |  |
|  |  | beach chair |  |
|  |  | fork |  |
|  |  | life ring |  |
|  |  | outlet |  |
|  |  | shovel |  |
|  |  | surfboard |  |
|  |  | clipboard |  |
|  |  | globe |  |

**Table 2.** Visual object pairs (object 1 + object 2) for Experiment 1b. All listed items were used as old items, new items were the same as those in Experiment 1a (see Table 1).

| **Object Relatedness** | **Object 1** | **Object 2** |
| --- | --- | --- |
| Related | drum | guitar |
|  | stapler | tape |
|  | key | coins |
|  | chair | door |
|  | xylophone | maracas |
|  | filing cabinet | printer |
|  | wolf | deer |
|  | snake | rat |
|  | laptop | smartphone |
|  | hammer | anvil |
|  | bell | clock |
|  | goat | pig |
|  | billiards | arcade game |
|  | cup straw | soda can |
|  | basketball | tennis racket |
|  | cup | teapot |
|  | cow | hen |
|  | kettle | blender |
|  | sword | bow arrow |
|  | spray bottle | sink |
|  | skateboard | bicycle |
|  | fireplace | axe |
| Unrelated | bat | ping pong paddle |
|  | bear | chick |
|  | dog | harmonica |
|  | scissors | wine bottle |
|  | hairdryer | cat |
|  | microwave | penguin |
|  | tiger | toilet |
|  | light switch | frog |
|  | saw | book |
|  | baseball bat | matches |
|  | keyboard | car |
|  | horse | camera |
|  | leopard | jet |
|  | goose | cymbal ride |
|  | elephant | boat |
|  | bird | drill |
|  | toaster | golfclub |
|  | motorcycle | pencil |
|  | flute | train |
|  | lighter | bowling pin |
|  | crocodile | piano |
|  | helicopter | toilet brush |
|  | washer | sled |

**Table 3.** Visual Stimuli for Experiment 2 and their Environment Pairs.

| **Environments** | **Object 1** |
| --- | --- |
| Farm (barn, pond) | anvil |
|  | basketball |
|  | bat |
|  | bear |
|  | bell |
|  | bicycle |
|  | bird |
|  | boat (toy) |
|  | bow arrow |
|  | broom |
|  | camera |
|  | car |
|  | cat |
|  | chainsaw |
|  | chick |
|  | coins |
|  | cow |
|  | cup |
|  | cup & straw |
|  | deer |
|  | drill |
|  | frog |
|  | goat |
|  | goose |
|  | guitar |
|  | hammer |
|  | harmonica |
|  | hen |
|  | horse |
|  | key |
|  | lighter |
|  | matches |
|  | motorcycle |
|  | pig |
|  | rat |
|  | saw |
|  | sheep |
|  | shovel |
|  | snake |
|  | soda can |
|  | tennis racket |
|  | train (toy) |
|  | wolf |
|  | wrench |
| House (living room, bedroom) | arcade game |
|  | baseball bat |
|  | billiards |
|  | blender |
|  | bowling pin |
|  | clock |
|  | crocodile (toy) |
|  | cymbal |
|  | dog |
|  | drum |
|  | elephant (toy) |
|  | filing cabinet |
|  | flute |
|  | golfclub |
|  | hairdryer |
|  | helicopter (toy) |
|  | jet (toy) |
|  | kettle |
|  | keyboard |
|  | knife |
|  | laptop |
|  | leopard (toy) |
|  | light switch |
|  | maracas |
|  | microwave |
|  | pencil |
|  | penguin (toy) |
|  | piano |
|  | ping pong paddle |
|  | printer |
|  | scissors |
|  | skateboard |
|  | sled |
|  | smartphone |
|  | stapler |
|  | sword |
|  | tape |
|  | teapot |
|  | tiger (toy) |
|  | toaster |
|  | wall alarm |
|  | washer |
|  | wine bottle |
|  | xylophone |

**Table 4**. Debriefing questions for Experiments 1a, 1b, and 2

| Experiment | Question | Response Options |
| --- | --- | --- |
| 1a, 1b, 2 | Was the volume on your computer enabled throughout the entire first task? | Yes; No |
|  | Did you adjust your volume at any time during the experiment? | Yes; No |
|  | How would you describe the volume of the sounds during the first task? | Quiet; Loud; Just right; I did not hear sounds |
|  | Did you use external speakers, in-ear headphones, over-ear headphones? | External speakers; In-ear headphones; Over-ear headphones; Other; I did not hear sounds |
|  | How would you describe the environment in which you completed the study? | Quiet; Mostly quiet; Somewhat noisy; Very noisy |
|  | Did you experience distractions during the study? | Yes, major distractions; Yes, minor distractions; No |
|  | How much effort did you put into the experiment? | Not any; Not very much; Some effort; A lot of effort |
|  | How difficult did you find task 1? | Not difficult; A little difficult; Very difficult |
|  | How difficult did you find task 2? | Not difficult; A little difficult; Very difficult |
|  | In the first task, did you ever experience a lag or gap between when the picture was shown and when the sound started? | Yes, a couple of times; Yes, often or always; No; I did not hear sounds |
| 1a, 1b | In the second task, did you understand when you were supposed to press the “Recollect” button? | Yes, definitely; Yes, I think so; Not sure; Not at all |
|  | Please give an example of something you recalled about an object on a trial where you pressed the “Recollect” button. | Free response |
| 2 | Give an example of something that helped you remember items better in the memory test. | Free response |
|  | Did you find it difficult to remember which environment you saw each item? | Not difficult; A little difficult; Very difficult |
|  | Did you find it difficult to remember where in the environment you saw each item? | Not difficult; A little difficult; Very difficult |
|  | Were there any tasks or parts of the study that you put less effort into? Briefly explain. | Free response |
|  |  |  |

**Table 5.** Experiment 1b additional results. Mean accuracy (% correct recognitions of old items) for Experiment 1b for items that were encoded as part of related and unrelated pairs for each Object Condition.

| Object Condition | Related | Unrelated | |
| --- | --- | --- | --- |
| Audiovisual Congruent | 78.85(17.04) | 72.41(15.01) |  |
| Visual Neighbor | 76.19(15.02) | 71.30(16.53) | |
| Audiovisual Control | 76.95(18.25) | 72.42(16.40) | |

Standard deviations are shown in parentheses.

*
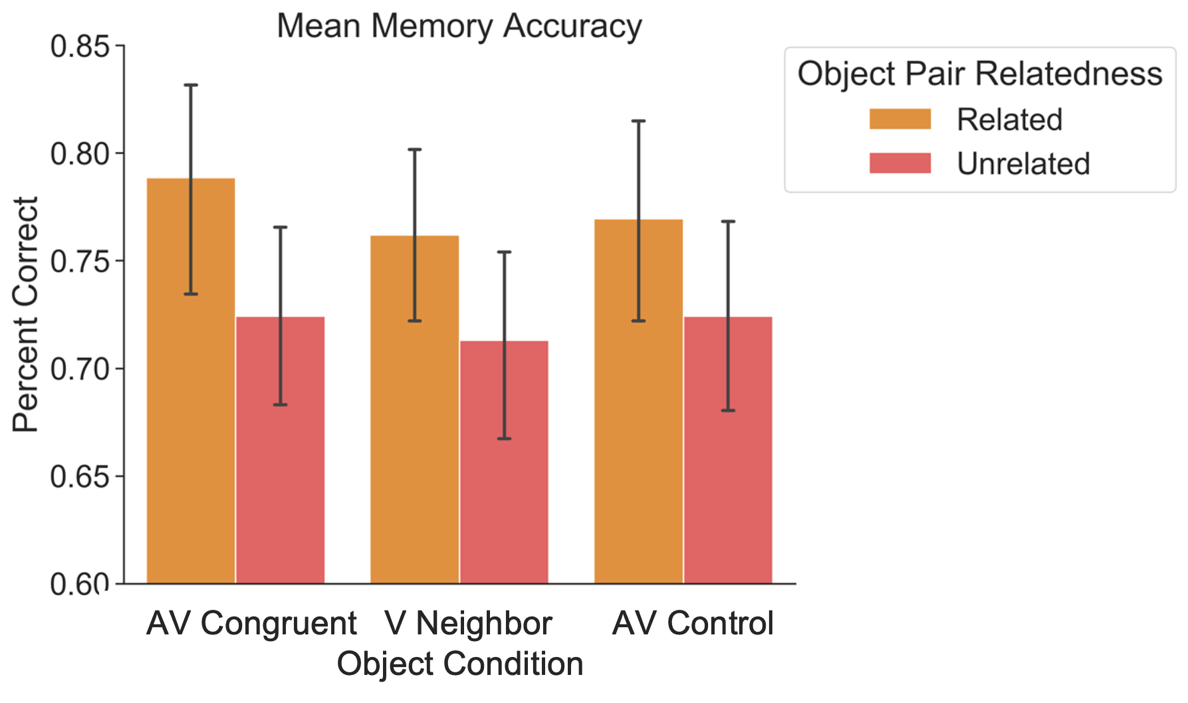
*

**Figure 1.** Accuracy (% correct recognitions of old items) for Experiment 1b for items that were encoded as part of related and unrelated pairs for each Object Condition. For a list of related and unrelated pairs, see Table 1. Error bars denote standard error of the mean.


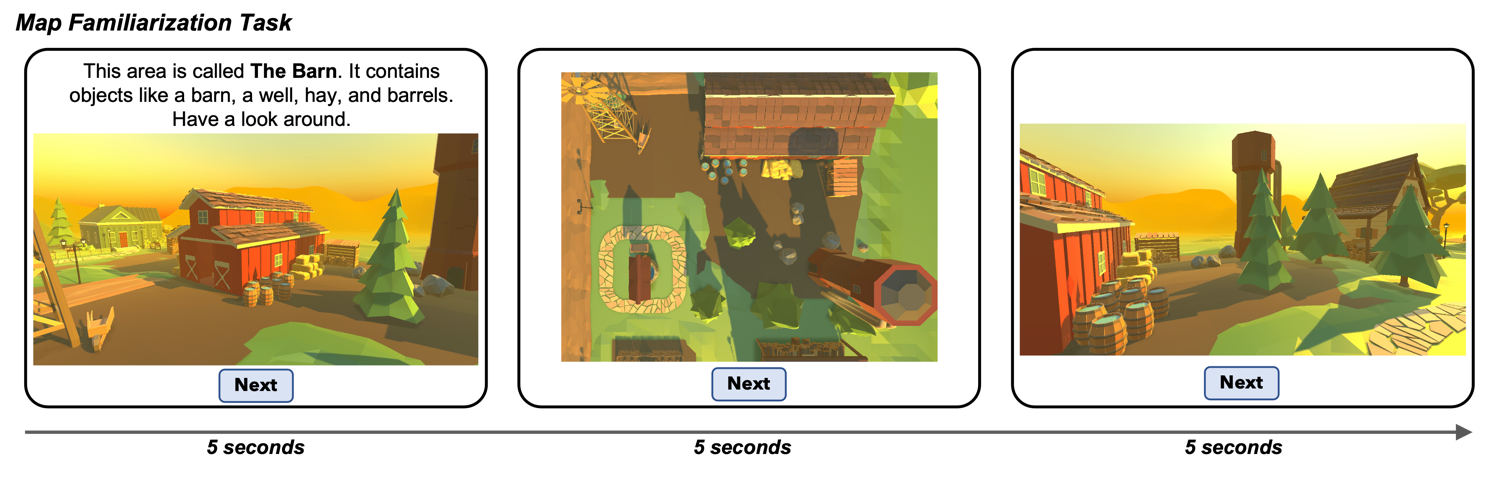


**Figure 2.** Map Familiarization task used in Experiment 2. Participants were introduced to the environments with written descriptions, overhead maps, and images of each environment. Each image was presented for five seconds, after which participants could advance at their own pace. They were instructed to familiarize themselves with these environments because they would be used in subsequent tasks.

**Table 6.** Demographic information for the entire sample including excluded participants for each experiment.

| Experiment | Total Participants | Mean Age | Female Count | | Male Count | Nonbinary Count |
| --- | --- | --- | --- | --- | --- | --- |
| 1a | 109 | 19.27 | 77 |  | 29 | 3 |
| 1b | 83 | 19.83 | 65 | | 17 | 1 |
| 2 | 209 | 19.35 | 152 | | 52 | 5 |

**Table 7.** Race and Ethnicity information for the entire sample including excluded participants for each experiment.

| Experiment | Racial Category | Hispanic/Latino | Not Hispanic/Latino | Total |
| --- | --- | --- | --- | --- |
| 1a | American Indian/Alaska Native | 1 | 0 | 1 |
|  | Asian | 0 | 61 | 61 |
|  | Native Hawaiian or Other Pacific Islander | 1 | 2 | 3 |
|  | Black or African American | 0 | 2 | 2 |
|  | White | 7 | 16 | 23 |
|  | More Than One Race | 2 | 4 | 6 |
|  | Not Listed | 12 | 1 | 13 |
| 1b | American Indian/Alaska Native | 1 | 0 | 1 |
|  | Asian | 0 | 46 | 46 |
|  | Native Hawaiian or Other Pacific Islander | 0 | 0 | 0 |
|  | Black or African American | 0 | 0 | 0 |
|  | White | 9 | 12 | 21 |
|  | More Than One Race | 3 | 1 | 4 |
|  | Not Listed | 10 | 1 | 11 |
| 2 | American Indian/Alaska Native | 0 | 0 | 0 |
|  | Asian | 2 | 130 | 132 |
|  | Native Hawaiian or Other Pacific Islander | 0 | 1 | 1 |
|  | Black or African American | 0 | 6 | 6 |
|  | White | 12 | 27 | 39 |
|  | More Than One Race | 5 | 9 | 14 |
|  | Not Listed | 15 | 2 | 17 |

| Experiment | | Task | Trial Type (Condition) | | Performance (% Correct) |
| --- | --- | --- | --- | --- | --- |
| 1a | Retrocue Size Judgement Task | | Overall | 60.27% (5.60%) | |
|  |  | | Audiovisual | 61.40% (7.19%) | |
|  |  | | Control sound | 58.00% (8.43%) | |
| 1b | Relational Judgement Task | | Overall | 75.93% (18.29%) | |
|  |  | | Audiovisual | 75.47% (17.92%) | |
|  |  | | Control sound | 76.40% (18.82%) | |
| 2 | Size Judgement Task | | Overall | 81.42% (9.68%) | |
|  |  | | Audiovisual | 81.85% (9.21%) | |
|  |  | | Control sound | 80.98% (10.17%) | |

**Table 8.** Encoding Performance in each experiment. Mean performance is reported, with standard deviations in parentheses. Overall mean performance is reported, as well as performance split between trials with meaningful sounds (Audiovisual) and control white noise sounds (Control sound)

**Appendix A: Remember/Know Analysis**

**Methods**

**Analysis Description**

To assess whether our ROC results converge with another common method of recollection and familiarity process dissociation, we included an explicit measure of recollection-based responses for Experiments 1a and 1b (the “recollect” response option) so that we could conduct an analysis in accordance with the remember/know (recollect/familiar) procedure (Tulving, 1985). For this, we assessed recollection/remember accuracy under a threshold assumption by subtracting incorrect “recollect” responses from correct “recollect” responses. We did this for each participant and each Object Condition, and compared recollection performance between Object Conditions using repeated measures analyses of variance (RM ANOVAs) for each experiment and post-hoc pairwise t-tests for significant F-tests. To assess familiarity/know-based recognition, we combined responses to the “definitely old,” “probably old,” “maybe old,” as “old” responses and combined “definitely new,” “probably new,” “maybe new,” as “new” responses. We used these values to compute the hit rates (the proportion of correct “old” responses to old items out of the total number of old items) for each participant and each Object Condition, and the false alarm rates (the proportion of incorrect “old” responses to new items out of the total number of new items) for each participant. We then calculated d-prime for each participant and each Object Condition by subtracting the z-scored false alarm rate from the z-scored hit rates, and then we performed RM ANOVAs for each experiment to compare familiarity-based recognition (d-prime) between Object Conditions.

**Results**

Experiment 1a: A RM ANOVA showed a significant effect of Object Condition on recollection (remember) responses, *F*(2, 98) = 6.21, *p* = 0.005, *η^2^* = 0.015, with more recollect responses for items in the Audiovisual Congruent condition than in the Audiovisual Control condition *t*(49) = 3.47, *p* = 0.003, and in the Neighboring Visual than in the Audiovisual Control Condition *t*(49) = 2.86, *p* = 0.02, but no significant difference between items in the Audiovisual Congruent and Neighboring Visual Conditions, *t*(49) = 1.02, *p* = 0.94. A RM ANOVA showed no significant effect of Object Condition on familiarity (know/d-prime) *F*(2, 98) = 0.70, *p* = 0.49.

Experiment 1b: A RM ANOVAs showed no significant effects of Object Condition on either recollection (remember) responses, *F*(2, 98) = 1.70, *p* = 0.19, or familiarity (know/d-prime), *F*(2, 98) = 0.52, *p* = 0.60.

These patterns of results converge with the ROC analyses reported in the article, with the exception of the significant difference in this remember/know analysis for Experiment 1a between the objects in the Visual Neighbor and Audiovisual Control Conditions. This provides evidence that there is a subjective difference between memories for objects in these conditions, even though this is not reflected in the recollection metric (*y-intercept*) in the ROC analysis for Experiment 1a.
